# The Waste of Clinical Material in Poor-Law Infirmaries

**Published:** 1912-05-11

**Authors:** 


### THE WASTE OF CLINICAL MATERIAL IN POOR-LAW INFIRMARIES.

BY A HOSPITAL SECRETARY.

The *Times* of last week raised a question which affects the very basis, both of the voluntary hospital system and of medical education as carried on in this country. The objects and aims of both sides are diametrically opposed. The interests of the school are to obtain good clinical material and a well-equipped teaching establishment: the interests of the charity are to serve solely the sick and needy. It says much for the forbearance and integrity of both sides that the system has been maintained. Our present method of relief in sickness for the indigent and very poor is divided in such a way that only a small moiety of those who have to seek free medical relief are at the disposal of the medical profession for purposes of medical education. Many thousands of sick poor occupy beds in the Poor-Law infirmaries, and they are by law, and by prejudice, unavailable for clinical purposes. So it comes about that a minority of the sick poor are at the disposal of the great teachers in medicine and surgery, and, oddly enough, this minority is to be found among those who are receiving care and treatment and are being maintained out of moneys provided for their charitable relief.

In most other countries every poor person unable to pay for medical treatment is required to be at the disposal of the medical profession for the purpose of medical education, and if that were the case here, the amount of clinical material would not be so restricted as it is at present. The public and the profession would benefit while the patients themselves would receive the best treatment, for it is admitted that the presence of a medical school attached to a hospital conduces to the welfare of the inmates.

It is only a short time ago that the President of the Local Government Board opened a hospital near London, with 800 beds, for sick children. In this most admirable institution are segregated the sick and injured children from the Poor-Law infirmaries of the Metropolis. This is the finest and largest hospital for children in the kingdom, and probably in Europe, and if the leaders of the medical profession had access thereto, would provide one of the best cliniques that any country could afford; but the medical profession, as a consultative teaching body, is excluded and the clinical material is lost. There can be no reason why in like manner various forms of disease should not be segregated from out the Poor-Law infirmaries. From the thousands of cases in the Union infirmaries could be culled material of the deepest interest.

Until the barrier is broken down and the medical profession afforded an opportunity of examining and teaching upon the cases under the Poor Law, so long will the difficulty exist between the medical schools and the voluntary hospitals. If the patients under the Poor Law were made available for medical education a wider field would be opened to the medical schools: there would no longer exist the absolute necessity for admitting to the voluntary hospitals cases of interest for clinical teaching, the wards of these institutions would be retained solely for the treatment of the sick and deserving poor, while there would not be the same incentive to spend charity money on medical research.

